# Antiproliferative Activity of (-)-Rabdosiin Isolated from *Ocimum sanctum* L.

**DOI:** 10.3390/medicines6010037

**Published:** 2019-03-12

**Authors:** Alexandros Flegkas, Tanja Milosević Ifantis, Christina Barda, Pinelopi Samara, Ourania Tsitsilonis, Helen Skaltsa

**Affiliations:** 1 Department of Pharmacognosy and Chemistry of Natural Products, Faculty of Pharmacy, National and Kapodistrian University of Athens, Panepistimiopolis, Zografou, 15771 Athens, Greece; alexflegas@yahoo.com (A.F.); kgtanja@yahoo.com (T.M.I.); cbarda@pharm.uoa.gr (C.B.); 2 Department of Biology, National and Kapodistrian University of Athens, Panepistimiopolis Zografou, 15784 Athens, Greece; psamara@biol.uoa.gr (P.S.); rtsitsil@biol.uoa.gr (O.T.)

**Keywords:** *Ocimum sanctum*, Lamiaceae, (-)-rabdosiin, cytotoxic activity, triterpenoids, phenolic derivatives

## Abstract

**Background:***Ocimum sanctum* L. (holy basil; Tulsi in Hindi) is an important medicinal plant, traditionally used in India. **Methods:** The phytochemical study of the nonpolar (dichloromethane 100%) and polar (methanol:water; 7:3) extracts yielded fourteen compounds. Compounds **6**, **7**, **9**, **11**, **12**, and **13**, along with the methanol:water extract were evaluated for their cytotoxicity against the human cancer cell lines MCF-7, SKBR3, and HCT-116, and normal peripheral blood mononuclear cells (PBMCs). **Results:** Five terpenoids, namely, ursolic acid (**1**), oleanolic acid (**2**), betulinic acid (**3**), stigmasterol (**4**), and *β*-caryophyllene oxide (**5**); two lignans, i.e., (-)-rabdosiin (**6**) and shimobashiric acid C (**7**); three flavonoids, luteolin (**8**), its 7-*O*-*β*-D-glucuronide (**9**), apigenin 7-*O*-*β*-D-glucuronide (**10**); and four phenolics, (*E)*-p-coumaroyl 4-*O*-*β*-D-glucoside (**11**), 3-(3,4-dihydroxyphenyl) lactic acid (**12**), protocatechuic acid (**13**), and vanillic acid (**14**) were isolated. Compound **6** was the most cytotoxic against the human cancer lines assessed and showed very low cytotoxicity against PBMCs. **Conclusions:** Based on these results, the structure of compound **6** shows some promise as a selective anticancer drug scaffold.

## 1. Introduction

Indigenous to India and parts of North and Eastern Africa, China, Hainan Island, and Taiwan, Tulsi (*Ocimum sanctum* L.; syn. *Ocimum tenuiflorum* L.) is referred to as “the elixir of life” or “the queen of herbs” and is believed to promote longevity [1,2]. Various parts of the plant are used in Ayurveda and Siddha traditional medicine to treat coughs, bronchitis, fever, bile disturbances, and has been also used as an anthelminthic, antiemetic, anticancer, antiseptic, antioxidant, antidiabetic anti-inflammatory, antiulcer, hepatoprotective, cardioprotective, anticoagulant, anticataract, and analgesic agent. Additionally, it has been reported that extracts of the plant can serve as vitalizers and rejuvenators, and are thought to increase life-expectancy and promote disease-free living [3,4,5,6,7,8,9,10,11,12,13,14,15,16,17]. 

Despite its wide therapeutic range, special care should be taken in case of the use of Tulsi in conjunction with other prescribed medicines since it exhibits various drug interactions. For example, its concomitant use with anticoagulants, such as heparin, warfarin, aspirin, clopidogrel, etc., is contraindicated due to allergic reactions that may occur. In addition, Tulsi increases the activity of phenobarbital and consequently may stimulate uterine contractions; thus, its use during pregnancy and lactation is not recommended [18,19].

The genus *Ocimum* L. is abundant in methylated flavones of the apigenin and luteolin types: cirsimartin, cirsilineol, isothymusin, and isothymonin. Terpenes such as triterpenic acids, ursolic, oleanolic acids, the oxygenated monoterpene carvacrol, the sesquiterpene hydrocarbon caryophyllene, the phenylpropenes eugenol and its methyl ether, as well as caffeic and rosmarinic acid are also present in significant amounts s. According to literature data, *O. sanctum* contains flavonoids, phenolics, neolignans, tannins, triterpenoids, sterols, cerebrosides, alkaloids, and saponin; most of them are well known for their in vitro and in vivo biological activities, such as antioxidant or prooxidant, cytotoxic, antitumor, anticarcinogenic, hepatoprotective, anti-inflammatory, as well as antiviral [3,4,5,6,19,20,21,22,23]. Moreover, the essential oil of *O. sanctum* contains high amount of eugenol (70%), also known for its antioxidant, anti-inflammatory, antimicrobial, and cytotoxic activities [24,25].

Based on the above, the plant is of high pharmacological importance, although it is still not fully chemically investigated. In this study, we analyzed both nonpolar and polar extracts of *O. sanctum* and studied the cytotoxic activity of its secondary metabolites.

## 2. Materials and Methods 

### 2.1. Plant Material

Aerial parts of *O. sanctum* L. were collected in flowering stage at Suriname, as previously described [21]. A voucher specimen (ATHS 093) has been deposited in the Herbarium of the Laboratory of Pharmacognosy, National and Kapodistrian University of Athens.

### 2.2. General Experimental Procedures

^1^H, ^13^C, and 2D NMR spectra were recorded in CDCl_3_ and CD_3_OD on Bruker DRX 400 and Bruker AC 200 (50.3 MHz for ^13^C NMR) instruments at 295 K. Chemical shifts are given in ppm (*δ*) and were referenced to the solvent signals at 7.24/3.31 and 77.0/49.0 ppm for ^1^H-/^13^C-NMR, respectively. COSY, HSQC, HMBC, HSQC-TOCSY (Heteronuclear Single Quantum Coherence-Total Correlation Spectroscopy), NOESY, and ROESY (Rotating-frame nuclear Overhauser Effect correlation SpectroscopY; mixing time 950 ms) were performed using standard Bruker microprograms. The solvents used were of spectroscopic grade (Merck). The [α]${}_{D}^{20}$ values were obtained in CHCl_3_ or MeOH on a Perkin-Elmer 341 Polarimeter. FT-IR spectra were recorded on a Perkin Elmer PARAGON 500 spectrophotometer. UV spectra were recorded on a Shimadzu UV-160 A spectrophotometer according to Mabry et al. (1970) [26]. GC–MS analyses were performed on a Hewlett-Packard 5973–6890 system operating in EI mode (70 eV) equipped with a split/splitless injector (220 °C), a split ratio 1/10, using a fused silica HP-5 MS capillary column (30 m x 0.25 mm (i.d.), film thickness: 0.25 μm) with a temperature program for HP-5 MS column from 60 °C (5 min) to 280 °C at a rate of 4 °C/min and helium as a carrier gas at a flow rate of 1.0 mL/min. Vacuum liquid chromatography (VLC): silica gel 60H (Merck, Art. 7736) [27]. Column chromatography (CC): silica gel (Merck, Darmstadt, Germany, Art. 9385), gradient elution with the solvent mixtures indicated in each case. Preparative thin layer chromatography (pTLC) was performed on silica gel (Merck, Art. 5721) and cellulose (Merck, Art. 5716). MPLC (Medium Pressure Liquid Chromatography) support: reversed-phase column (Μerck, 10167): 36 × 3.6 cm (Büchi Borosilikat 3.3, Code 19674), 24 × 1.5 cm (Büchi Borosilikat 3.3, Code 2813) on a system (Büchi Pump C-615). HPLC (High Performance Liquid Chromatography) support: preparative HPLC was performed using (a) Kromasil 100 si Semi-prep 25 cm × 10 mm and (b) Kromasil C_18_ 25 cm × 10 mm columns on a HPLC system (Jasco PU-2080) equipped with a RI detector (Shimadzu 10 A). Fractionation was always monitored by TLC silica gel 60 F-254, (Merck, Art. 5554) with visualization under UV (254 and 365 nm) and spraying with vanillin–sulfuric acid reagent (vanillin Merck, Art. No. S26047 841) and with Neu’s reagent for phenolics [28].

### 2.3. Extraction and Isolation

The initial extraction was previously described [21]. In brief, the aerial parts of *O. sanctum* L. (0.40 kg) were air-dried and finely ground, and then extracted at room temperature using dichloromethane and methanol, successively. 

Part of the dichloromethane residue (11.9 g) was re-extracted at room temperature with ethyl acetate (EtOAc) and *n*-BuOH, yielding two fractions (A and B). Fraction A (7.8 g) was fractionated by VLC on silica gel using mixtures of cyclohexane and EtOAc of increasing polarity (100:0; 90:10; 80:20; 70:30; 60:40; 50:50; 40:60; 30:70) and yielded 8 subfractions (A_1_–A_8_). Subfractions A_3_ (eluted with cyclohexane:EtOAc 80:20) and A_4_ (eluted with cyclohexane:EtOAc 70:30) were combined to group AA (401.7 mg), subjected to CC over silica gel using mixtures of cyclohexane and EtOAc and yielded 81 fractions combined to 11 groups (AA_1_–AA_11_). Purification on preparative TLC of fraction AA_3_ (51.8 mg; eluted with cyclohexane:EtOAc 95:5) yielded compound **5** (1.3 mg). Fractions AA_6_ (34.7 mg; eluted with cyclohexane:EtOAc 97:3) and AA_8_ (34.4 mg; eluted with cyclohexane:EtOAc 85:15) were further fractionated by normal-phase HPLC (isocratic elution cyclohexane:EtOAc 75:25) and yielded compounds **4** (*t_R_* 21.84 min; 3.2 mg), **2** (*t_R_* 16.01 min; 1.7 mg), and **3** (*t_R_* 14.84 min; 5.5 mg). Fraction B purified by CC on silica gel using mixtures of cyclohexane and EtOAc yielded 131 fractions combined to 18 groups (B_1_–B_18_). Fraction B_5_ (eluted with cyclohexane:EtOAc 80:20) was identified as compound **1** (1.8 mg)**,** while fraction B_8_ (eluted with cyclohexane:EtOAc 70:30) as compound **14** (2.3 mg). 

Part of the methanol residue (3.6 g) was subjected to RP_18_-MPLC using a H_2_O:MeOH gradient system (100:0; 90:10; 85:15; 80:20; 75:25; 50:50; 0:100; 0:100; 50 min each) and yielded 8 fractions (M_1_-M_8_). Group M_2_ (eluted with H_2_O:MeOH 90:10) was applied to CC on silica gel with mixtures of dichloromethane:methanol:water of increasing polarity to give 151 fractions (combined to 14 groups; M_2-1_–M_2-14_) and afforded compounds **13** (M_2-5_ eluted with DM:MeOH: H_2_O 95:5:0.3; 40.5 mg), **11** (M_2-11_ eluted with DM:MeOH:H_2_O 70:30:3; 1.6 mg), and **12** (M_2-12_; 4.3 mg; eluted with DM:MeOH:H_2_O 40:60:6). M_3_ (290.0 mg) was further purified on Sephadex LH-20 eluted with MeOH (100%) and yielded 30 fractions combined in 10 subfractions (M_3-1_–M_3-10_). M_3-6_ (57.0 mg) was subjected to reversed-phase HPLC (isocratic elution; methanol:AcOH 5% 7:3) to give compounds **6** (*t_R_* 23.90 min; 7.5 mg), **9** (*t_R_* 29.30 min; 1.9 mg), and **7** (*t_R_* 35.20 min; 3.7 mg). M_6_ (674.2 mg) was similarly fractionated by CC over silica gel with mixtures of CH_2_Cl_2_:MeOH:H_2_O of increasing polarity and yielded 135 fractions combined in 25 subgroups (M_6-1_–M_6-25_). Subgroup M_6-24_ (eluted with CH_2_Cl_2_:MeOH:H_2_O 70:30:3; 69.4 mg) was subjected to CC on silica gel as previously described to give 75 fractions; fraction 8 (1.3 mg) was identified as compound **10**. Another part of the methanol extract (7.7 g) was redissolved in water and extracted at room temperature with EtOAc and *n*-BuOH, affording three fractions (MA-MC). MB (eluted with *n*-BuOH; 5.3 g) was subjected to RP_18_-MPLC using a H_2_O:MeOH gradient system (100% H_2_O→100% MeOH; steps of 10% MeOH) and yielded 11 fractions (MB_1_-MB_10_). Fraction MB_3_ (eluted with H_2_O:MeOH 80:20) was identified as compound **8** (13.6 mg).

It is notable that during the fractionation and isolation procedures, all extracts and subfractions were continuously monitored by analytical TLC and ^1^H-NMR. All obtained fractions were concentrated to dryness under vacuum (30 °C) and placed in activated desiccators with P_2_O_5_ until their weights were stabilized.

### 2.4. Cytotoxic Effects against Cancer Cell Lines

The cytotoxic activity of the compounds, as well as of the initial methanol extract, were tested against three human cancer cell lines: MCF-7 (breast; estrogen receptor positive (ER+), progesterone receptor (PR)+, and HER2 negative (-)), SKBR3 (breast; ER-, PR-, and HER2+), and HCT-116 (colon). All cell lines were maintained in RPMI-1640, supplemented with 10% heat-inactivated fetal bovine serum (FBS), 10 mM Hepes, 10 U/mL penicillin, 10 U/mL streptomycin, and 5 mg/mL gentamycin (all from Lonza, Cologne, Germany) (thereafter referred to as complete medium) at 37 °C in a humidified 5% CO_2_ incubator.

Compounds were prepared at a stock solution of 10.0 mg/mL in DMSO and the extract at 20.0 mg/mL in DMSO. Prior to their use, they were diluted in plain RPMI-1640. Cytotoxicity was evaluated by the MTT reduction assay [29], which determines the effect of treatment with an exogenously added agent on the viability of the cell population. Briefly, cells were plated in 96-well plates (Greiner Bio-One GmbH, Frickenhausen, Germany; 5 × 10^3^ cells/well) and incubated at 5% CO_2_ and 95% air at 37 °C for 24 h, in order to adhere. Further, cells were incubated with the compounds for 72 h at 37 °C in a 5% CO_2_ incubator. The MTT reagent (Sigma-Aldrich, Darmstadt, Germany; 1 mg/mL in phosphate buffered saline (PBS); 100 μL/well) was added during the last 4 h of incubation. The formazan crystals formed were dissolved by adding 0.1 M HCl in 2-propanol (100 μL/well) and absorption was measured using an ELISA reader (Denley WeScan, Finland) at 545 nm with reference filter set at 690 nm. All cultures were set in triplicate, whereas cells incubated in complete medium or in medium containing the equivalent amount of DMSO, as well as cells incubated in the presence of doxorubicin (Sigma-Aldrich) were used as negative and positive controls, respectively. The half maximal inhibitory concentration (IC_50_) was calculated according to the formula: 100(A_0_ − A)/A_0_ = 50, where A and A_0_ are optical densities of wells exposed to the compounds and control wells, respectively.

The compounds were tested at a concentration range of 200.0 to 6.25 μg/mL and the extract at 750.0 to 1.25 μg/mL. Doxorubicin was used as a standard cytotoxic agent and showed IC_50_ values ≤ 0.2 μM in all cell lines tested. All experiments were performed at least three times. 

### 2.5. Flow Cytometry Analysis

MCF-7, SKBR3 and HCT-116 cells were incubated with compound **6** and analyzed with flow cytometry following staining with annexin V and propidium iodide (PI). Cells were plated into 24-well plates (Greiner Bio-One; 3 × 10^5^/mL; 2 mL/well), let adhere overnight, and incubated with the mean IC_50_ value (80 μg/mL) and 40 μg/mL of compound **6** for 72 h. Cells were detached with 2 mM EDTA in Dulbecco’s PBS (DPBS), harvested, centrifuged in cold PBS (1500 rpm; 5 min), and stained with the Annexin V-FITC Apoptosis Detection Kit (BioLegend, Fell, Germany; cat# 640914), according to the manufacturers’ instructions. In brief, cells were resuspended in binding buffer, then annexin V-FITC (5 μL) and PI (10 μL; 0.03 μg/sample) were added, mixed, and incubated with the cells for 15 min in the dark at room temperature. The volume was adjusted to 500 μL with binding buffer and the cell suspension was immediately analyzed in a FACSCanto II (BD Biosciences, San Diego, CA, USA) using FACSDiva software (V7, BD Biosciences).

### 2.6. Cytotoxic Effect against Human Peripheral Blood Mononuclear Cells

Compound **6** was additionally assessed for its cytotoxicity against human peripheral blood mononuclear cells (PBMCs) isolated from healthy blood donors’ peripheral blood as previously described [30]. Prior to blood draw, individuals gave their informed consent according to the regulations approved by the 2nd Peripheral Blood Transfusion Unit and Hemophiliac Centre, “Laikon” General Hospital Institutional Review Board, Athens, Greece. PBMCs were seeded in 24-well plates (5 × 10^5^/mL; 2 mL/well) and exposed to 2 concentrations of compound **6**: 80 μg/mL and 40 μg/mL. PBMCs were collected, stained as described in 2.5 and analyzed by flow cytometry. 

## 3. Results and Discussion

### 3.1. Secondary Metabolites Isolated from O. sanctum

The phytochemical study of both nonpolar and polar extracts from *Ο. sanctum* aerial parts led to the isolation of 14 compounds identified on the basis of their spectra. More specifically, five terpenoids, i.e., ursolic acid (**1**) [31], oleanolic acid (**2**) [32], betulinic acid (**3**) [32,33], stigmasterol (**4**) [33], and *β*-caryophyllene oxide (**5**) [34]; two lignans, (-)-rabdosiin (**6**) [35,36] and shimobashiric acid C (**7**) [37]; three flavonoids, luteolin (**8**) [38], its 7-*O*-*β*-d-glucuronide (**9**) [39,40,41], and apigenin 7-*O*-*β*-d-glucuronide (**10**) [42,43]; and phenolic compounds, (E)-*p*-coumaroyl 4-*O*-*β*-d-glucoside (**11**) [44], 3-(3,4-dihydroxyphenyl) lactic acid (**12**) [45], protocatechuic acid (**13**) [46], and vanillic acid (**14**) [46] were isolated. This is the first time that compounds **6**, **7**, **11**, and **12** were isolated from this plant.

According to the literature, the taxonomic description of the genus *Ocimum* L. is still debatable. It is composed of three subgenera, namely subgenus Ocimum (comprising three sections: Ocimum, Gratissima and Hiantia), subgenus Nautochilus, and subgenus Gymnocimum. The species (*O. sanctum* L.) under investigation has been located in the subgenus Gymnocimum. This subgenus can be distinguished because of the existence of flavonoid glucuronides, which are found in plants of the subgenera Nautochilus and Ocimum [38]. Consequently, our work is in agreement with previous studies regarding the chemical profile of the subgenus Gymnocimum. Moreover, it was previously shown that 3-(3,4-dihydroxyphenyl) lactic acid is a precursor of the nonenzymatic synthesis of (*S*)-(-)-rosmarinic acid and (+)-rabdosiin [47], therefore its identification (compound **12**) could be related to the biosynthesis of (-)-rabdosiin (**6**) [48].

**Figure 1:**
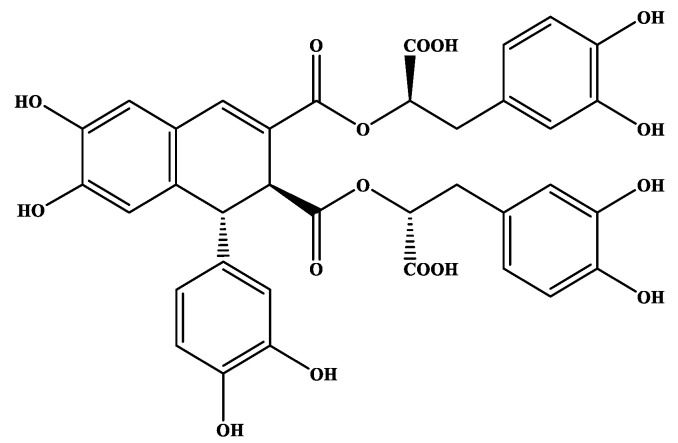
Chemical structures of (-)-rabdosiin (**6**) isolated from *O. sanctum*.

Compound (-)-rabdosiin (**6**) (Figure 1) is a caffeic acid tetramer connected to a lignan skeleton. Originally, it has been isolated and identified from the stem of *Rabdosia japonica*, Labiatae [35], while both enantiomers (-)-rabdosiin and (+)-rabdosiin were later isolated from *Macrotomia euchroma*, Boraginaceae [49] and also from other plants of this family such as *Lithospermum erythrorhizon* [50] and *Eritrichium sericeum* [36]. Based on the fact that the entire fractionation and isolation procedures were continuously monitored by ^1^H-NMR, the active compound **6** was not detected in other fractions (NMR data of **6** are provided as Supplementary Materials, Tables S1 and S2, Figures S1–S6). Consequently, being a minor compound of the plant, its activity could derive in synergy with other constituents. 

According to published data, rabdosiin and the similar caffeic acid derivatives have been suggested as potential anti-HIV and antiallergic agents. Moreover, studies showed that rabdosiin is an antioxidant factor (acting as an effective scavenger of reactive oxygen species), as well as a possible inhibitor of hyaluronidase and *β*-hexosaminidase release [51,52]. Nevertheless, to the best of our knowledge, the antiproliferative activity of rabdosiin is reported for the first time. 

### 3.2. Antiproliferative Activityod of Secondary Metabolites of O. sanctum

Using the MTT dye reduction assay, the methanol:water extract (7:3) and 6 purified secondary metabolites (compounds **6**, **7**, **9**, **11**, **12**, and **13**) were screened for their cytotoxic/cytostatic activity against human breast and colon cell lines. Our results showed that the extract was cytotoxic against all cell lines, with an IC_50_ range of 45 ± 2.12 to 57 ± 14.14 μg/mL (Table 1). Based on these data, we further proceeded to the screening of the isolated natural products **6**, **7**, **9**, **11**, **12**, and **13** against MCF-7 cells which was the mostly affected cell line exposed to the methanol extract of *Ο. sanctum* L. The IC_50_ values calculated are presented in Table 1. Among the purified compounds, the most prominent was **6**, which was further tested against SKBR3 and HCT-116 cells. Overall, compound **6** demonstrated a considerable cytotoxic activity, with IC_50_ values 75 ± 2.12, 83 ± 3.54 and 84 ± 7.78 μg/mL against MCF-7, SKBR3, and HCT-116, respectively. 

**Figure 2:**
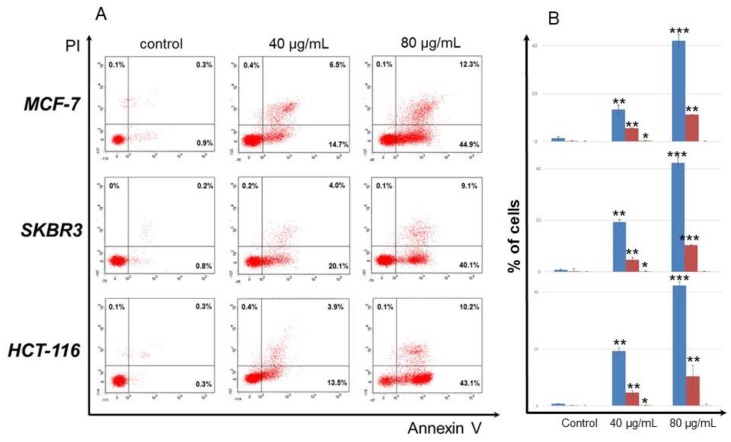
Compound **6** induced apoptosis to human cancer cells. MCF-7, SKBR3, and HCT-116 cells were exposed to 40 and 80 μg/mL of compound **6** for 72 h, stained with annexin V and PI, and analyzed by flow cytometry. Control cells were incubated in complete medium supplemented with 0.5% DMSO. Flow cytometry analysis was performed using FACS Diva software. (**A**). Representative dot plots from cells treated with compound **6**. Percentages of early apoptotic (lower right), late apoptotic/necrotic (upper right), and necrotic (upper left) are shown in each quadrat. (**B**). Histograms of apoptotic and necrotic cells after exposure to compound **6**. Blue columns show percentages of early apoptotic, red columns of late apoptotic and green columns of necrotic cells. Mean values ± SD from 3 experiments are shown. *, *p* < 0.05; **, *p* < 0.01; ***, *p* < 0.001, in all cases compared to control after Student’s unpaired *t*-tests.

To analyze the type of cell death (apoptosis or necrosis) induced by compound **6** on MCF-7, SKBR3, and HCT-116 cells, cells were stained with annexin V which binds phosphatidylserine exposed on the surface of apoptotic cells and PI which intracellulary stains the DNA of necrotic cells. As shown in Figure 2, 80 μg/mL of compound **6** drove ca. 50% of all cells to apoptosis. Specifically, 44.9% of MCF-7 were annexin V+ and 12.3% annexin V+/PI+, suggesting that cells exposed to compound **6** underwent early apoptosis and a small percentage thereof late apoptosis/necrosis. Analogous percentages were obtained for SKBR3 (40.1% early apoptotic; 9.1% late apoptotic/necrotic) and HCT-116 (43.1% early apoptotic; 10.2% late apoptotic/necrotic) cells. When the same cell lines were exposed to 40 μg/mL of compound **6**, the percentages of early apoptotic and late apoptotic/necrotic cells were reduced ca. by 50% (13.5–20.1% and 3.9–6.5%, respectively), suggesting that induction of apoptosis by compound **6** is concentration-dependent.

**Figure 3:**
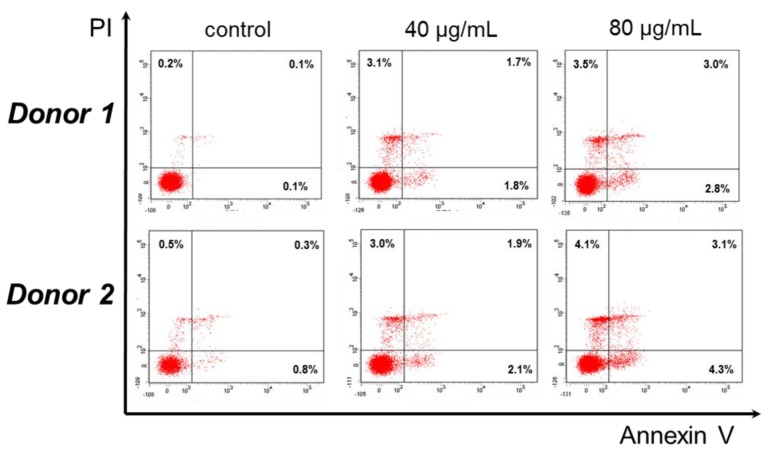
Compound **6** does not induce apoptosis or necrosis to peripheral blood mononuclear cells (PBMCs). PBMCs were isolated from 2 different donors (1 and 2) and incubated with 40 and 80 μg/mL of compound **6** for 24 h. Other details as in Legend of Figure 2. Representative dot plots from both donors are shown from one experiment performed in duplicate.

Based on the significant cytotoxic activity of compound **6** against cancer cell lines we further tested whether it may also be toxic against normal cells, i.e., PBMCs isolated from two different healthy blood donors. PBMCs were incubated for 24 h with the IC_50_ and the 1/2 concentration of **6**, stained and analyzed by flow cytometry. Interestingly, the IC_50_ of compound **6** (80 μg/mL) induced early and late apoptosis/necrosis in a small percentage of PBMCs (2.8% and 3.0% for donor 1; 4.3% and 3.1% for donor 2, respectively). At half concentration, the percentages were highly reduced and much less early apoptotic and late apoptotic/necrotic cells were detected (1.8% and 1.7% for donor 1; 2.1% and 1.9% for donor 2, respectively) (Figure 3).

The good antitumor activity of compound **6** against human cancer cells and the simultaneous marginal cytotoxicity of the same compound when tested against normal human cells (PBMCs), suggest that (-)-rabdosiin may display less toxic side effects when administered in vivo. In support of our results, the few studies carried out in the last decade on the potential anticancer activity of *O. sanctum* extracts and its essential oil with different human cancer cell lines, clearly suggest that Tulsi may be used as a supplement to enhance anticancer chemotherapy without causing severe damage to normal epithelial cells [25,53,54]. Botanical drugs are currently approved in therapy with specific indications and in the last decades, research has focused on the anticancer effect of plant extracts.

Taken altogether, (-)-rabdosiin displays an interesting proapoptotic activity against cancer cell lines and in parallel shows a noticeable selectivity to malignant cells. It is noteworthy that the cytotoxic response of the extract is better compared to the other isolated compounds, including compound **6**. As (-)-rabdosiin is a minor compound of the plant, we assume that it contributes to the improved antiproliferative activity of the methanol extract, and that it is probably synergistically with other active metabolites. The good activity of the polar extract, as well as of compound **6** against a series of human cancer cell lines and its marginal cytotoxicity against PBMCs, give evidence toward the effective use of this plant for the prevention of human cancer. Moreover, the core structure of (-)-rabdosiin could be considered as drug lead in anticancer drug design.

## Figures and Tables

**Table 1:** In vitro cytotoxicity of the methanol extract and isolated compounds from Tulsi on human cancer cell lines.

	IC_50_ ± SD (in μg/mL) ^a^							IC_50_ ± SD (in μΜ)
Compounds	6	7	9	11	12	13	Extract *	Doxorubicin
MCF-7	75 ± 2.12 ^a^	142 ± 3.54	141 ± 1.41	139 ± 7.78	140 ± 12.02	140 ± 4.95	45 ± 2.12	0.092 ± 0.007
SKBR3	83 ± 3.54	ΝΤ	ΝΤ	ΝΤ	ΝΤ	ΝΤ	46 ± 5.66	0.095 ± 0.008
HCT-116	84 ± 7.78	ΝΤ	ΝΤ	ΝΤ	ΝΤ	ΝΤ	57 ± 14.14	0.192 ± 0.029

* Methanol:water 70:30 ^a^ IC_50_ values were determined after 72 h of exposure to each compound and represent means ± standard deviation (SD) of three independent experiments performed; Doxorubicin was used as positive control and showed IC_50_ ≤ 0.20 μM for all cell lines assayed.

## Supplementary Materials

The following are available online at https://www.mdpi.com/2305-6320/6/1/37/s1, Table S1: ^1^Η-NMR of **6** (CD_3_OD, 400 MHz); Table S2: ^13^C-NMR of **6** (CD_3_OD, 400 MHz); Figure S1: ^1^H—NMR spectrum of **6** (CD_3_OD, 400 Hz); Figure S2: COSY spectrum of **6** (CD_3_OD, 400 Hz); Figure S3: ^13^C NMR spectrum of **6** (CD_3_OD, 400 Hz); Figure S4: HSQC spectrum of **6** (CD_3_OD, 400 Hz); Figure S5: HMBC spectrum of **6** (CD_3_OD, 400 Hz); Figure S6: Most important HMBC signals of compound **6**.
