# Supplementary material for: Antiproliferative Activity of (-)-Rabdosiin Isolated from Ocimum sanctum L
Source: Medicines (Basel). 2019 Mar 12;6(1):37. doi: 10.3390/medicines6010037 (PMC6473744; doi:10.3390/medicines6010037)
Supplement: Supplementary file 1 [file medicines-06-00037-s001.pdf]

Supplementary Material: Antiproliferative Activity of (-)-Rabdosiin Isolated from *Ocimum sanctum* L.

Alexandros Flegkas, Tanja Milosević Ifantis, Christina Barda, Pinelopi Samara, Ourania Tsitsilonis and Helen Skaltsa

Table 1. ^{1}H-NMR of **6** (CD_3OD, 400MHz).

δ (ppm)	n°H	J (Hz)	Interpretation
7.58	1	*s*	H-4
6.81	1	*s*	H-5
6.74	1	*d* (J = 2.0)	H-2''
6.68	1	*d* (J = 1.8)	H-2'''
6.67	1	*d* (J = 8.0)	H-5'''
6.66	1	*d* (J = 8.0)	H-5''
6.62	1	*d* (J = 8.2)	H-5'
6.58	1	*dd* (J = 8.0; 2.0)	H-6''
6.51	1	*dd* (J = 8.0; 2.0)	H-6'''
6.49	1	*s*	H-8
6.35	1	*dd* (J = 8.0; 2.0)	H-6'
6.32	1	*d* (J = 2.0)	H-2'
5.05	1	*dd* (J = 7.8; 4.4)	H-8'''
4.99	1	*dd* (J = 7.2; 5.2)	H-8''
4.44	1	br*s*	H-1
3.90	1	*d* (J = 2.0)	H-2
3.04	1	*dd* (J = 14.4; 4.2)	H-7'''a
2.98–2.88	2	*m*	H-7''a,b
2.93	1	*dd* (J = 14.0; 7.8)	H-7'''b

Table 2. ^{13}C-NMR of **6** (CD_3OD, 400MHz).

Position	*δ* (ppm)	Type of C
1	45.9	CH
2	48.1	CH
3	120.7	C
4	140.6	CH
5	116.8	CH
6	144.5	C
7	148.9	C
8	117.2	CH
9	131.0	C
10	124.2	C
1'	135.9	C
2'	115.2	CH
3'	144.1	C
4'	144.2	C
5'	115.8	CH
6'	119.6	CH
1''	128.9	C
2''	117.3	CH
3''	145.5	C
4''	144.4	C
5''	116.2	CH
6''	121.7	CH
7''	37.4	CH_2
8''	74.9	CH
9''	172.4	C=O
1'''	128.9	C
2'''	116.1	CH
3'''	145.5	C
4'''	144.4	C
5'''	116.7	CH
6'''	121.7	CH
7'''	37.4	CH_2
8'''	74.9	CH
9'''	173.3	C=O
2-COO	173.1	C=O
3-COO	167.8	C=O

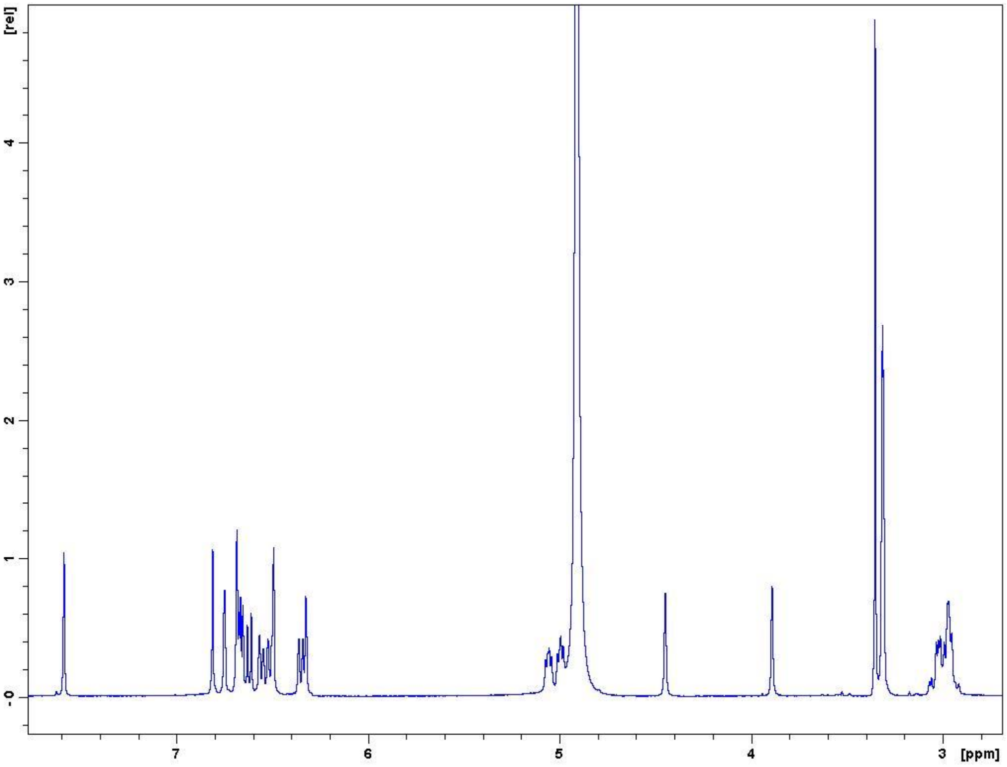

Figure 1. ^{1}H—NMR spectrum of **6** (CD_3OD, 400 Hz).

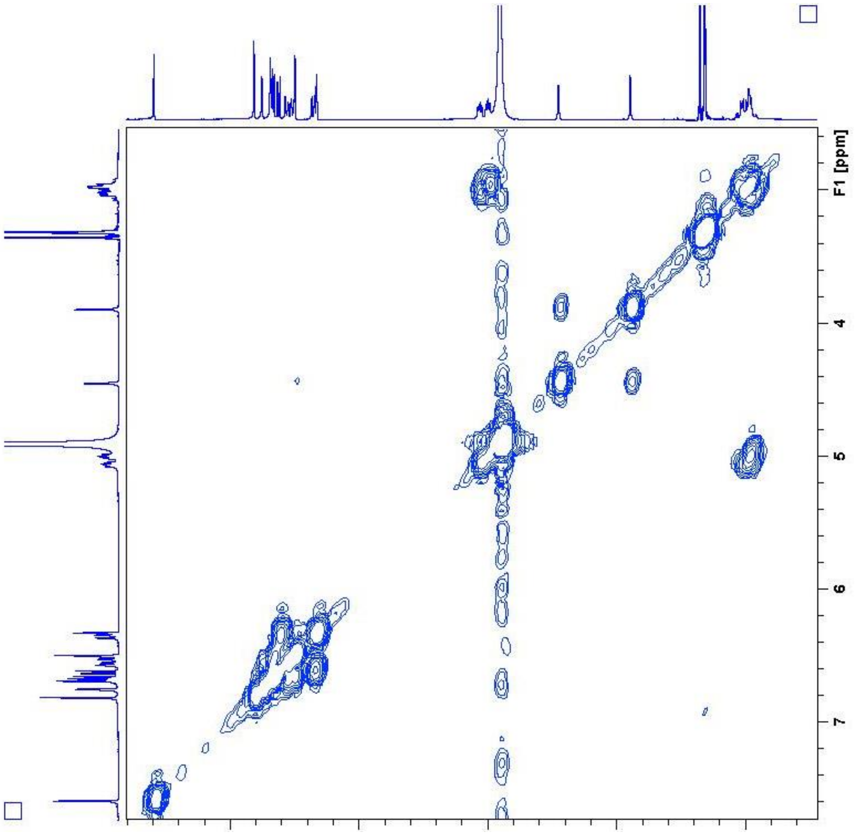

Figure 2. COSY spectrum of **6** (CD_3OD, 400 Hz).

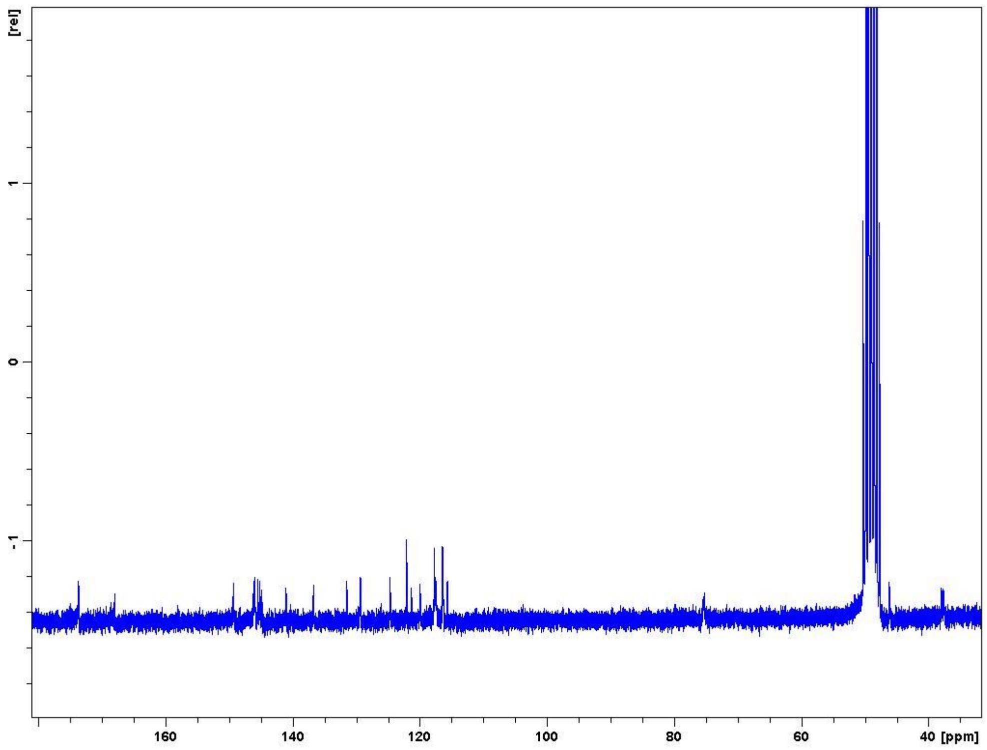

Figure S3. ^{13}C NMR spectrum of **6** (CD_3OD, 400 Hz).

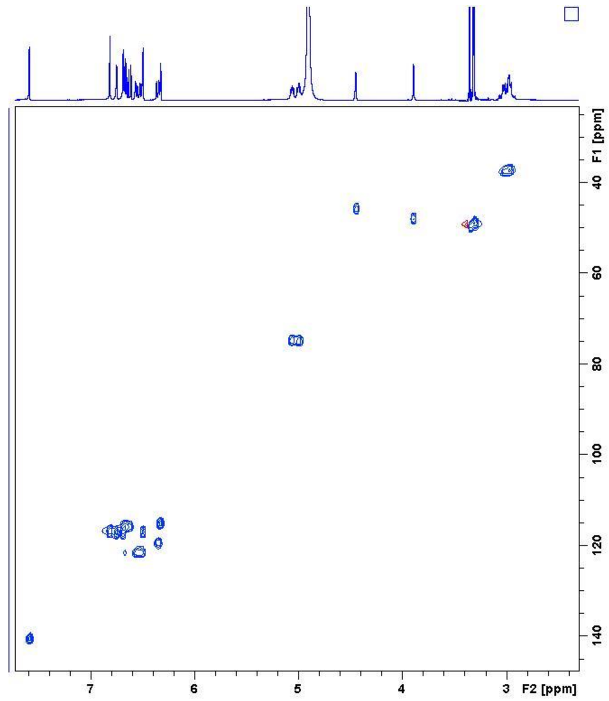

Figure 4. HSQC spectrum of **6** (CD_3OD, 400 Hz).

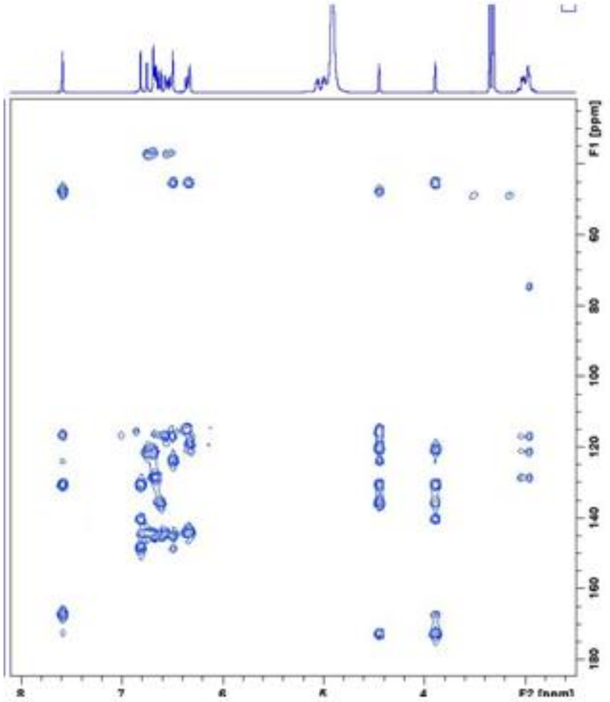

Figure 5. HMBC spectrum of **6** (CD_3OD, 400 Hz).

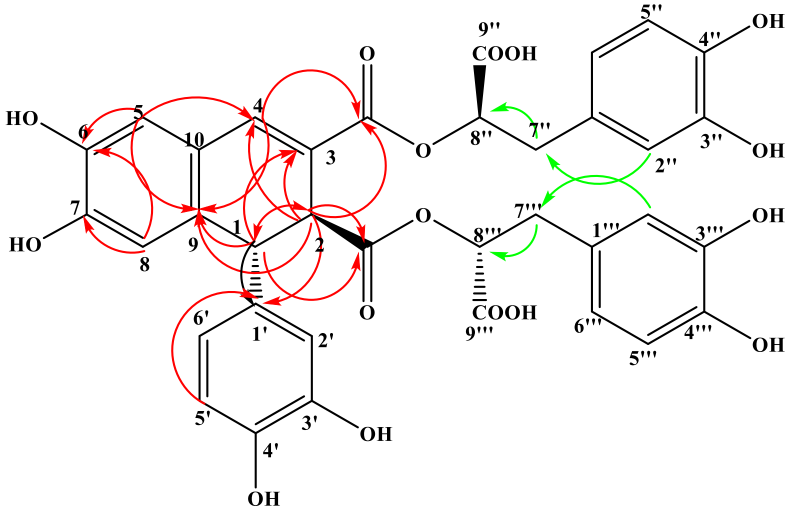

Figure 6. Most important HMBC signals of compound **6**.
